# Aldose Reductase B1 in Pig Seminal Plasma: Identification, Localization in Reproductive Tissues, and Relationship With Quality and Sperm Preservation

**DOI:** 10.3389/fcell.2021.683199

**Published:** 2021-06-08

**Authors:** Yentel Mateo-Otero, Estel Viñolas-Vergés, Marc Llavanera, Jordi Ribas-Maynou, Jordi Roca, Marc Yeste, Isabel Barranco

**Affiliations:** ^1^Biotechnology of Animal and Human Reproduction (TechnoSperm), Institute of Food and Agricultural Technology, University of Girona, Girona, Spain; ^2^Unit of Cell Biology, Department of Biology, Faculty of Sciences, University of Girona, Girona, Spain; ^3^Department of Medicine and Animal Surgery, Faculty of Veterinary Medicine, University of Murcia, Murcia, Spain; ^4^Department of Veterinary Medical Sciences, University of Bologna, Bologna, Italy

**Keywords:** aldose reductase B1, AKR1B1, seminal plasma, ejaculate fractions, sperm physiology, pig

## Abstract

Aldose reductase B1 (AKR1B1), a NADPH-dependent enzyme that belongs to the aldo-keto reductase protein superfamily, has been reported to be involved in both male and female reproductive physiology. The objectives of this study were: (1) to evaluate the concentration of SP-AKR1B1 in pig ejaculate fractions; (2) to describe the immunohistochemical localization of AKR1B1 alongside the boar genital tract; (3) to evaluate the relationship between SP-AKR1B1 and sperm quality/functionality parameters. Ejaculates from seven boars (one ejaculate per boar) were collected in separate portions [the first 10 mL of the sperm rich fraction (SRF-P1), the rest of the SRF (SRF-P2), and the post-SRF (PSRF)], and the concentration of SP-AKR1B1 was assessed using an enzyme-linked immunosorbent assay (ELISA). Immunohistochemistry and immunoblotting targeting was conducted in the reproductive tissues of these boars. Additionally, the entire ejaculates of 14 boars (one ejaculate per boar) were collected and split into three separate aliquots for: (i) SP-AKR1B1 quantification; (ii) assessment of sperm concentration and morphology; and (iii) evaluation of sperm quality and functionality parameters upon ejaculate collection (0 h) and after 72 h of liquid storage at 17°C. Concentration of AKR1B1 in the SP of SRF-P1 (458.2 ± 116.33 ng/mL) was lower (*P* < 0.05) than that of SRF-P2 (1105.0 ± 229.80 ng/mL) and PSRF (1342.4 ± 260.18 ng/mL). Monomeric and dimeric AKR1B1 forms were expressed alongside the reproductive tissues, except in the bulbourethral glands. No relationship between SP-AKR1B1 and sperm quality/functionality parameters was observed either at 0 h or after 72 h of storage at 17°C. In conclusion, AKR1B1 is expressed in the reproductive organs of boars (except bulbourethral glands) and a higher concentration is found in the PSRF suggesting that seminal vesicles would be the main secretory source. However, this enzyme does not appear to be related to sperm quality/functionality or to the sperm ability to withstand liquid storage at 17°C.

## Introduction

Aldose reductase B1 (AKR1B1 or ALR2), a NADPH-dependent enzyme that belongs to the aldo-keto reductase protein superfamily (Bohren et al., 1989; Hyndman et al., 2003), has been reported to play an essential role in both male and female reproductive systems in humans (Bresson et al., 2011), cattle (Frenette et al., 2004; Girouard et al., 2009), rats (Kobayashi et al., 2002), sheep (Yang et al., 2019), and pigs (Steinhauser et al., 2016; Pérez-Patiño et al., 2018). This enzyme is known to be involved in the polyol pathway for fructose production, specifically in the conversion of glucose into sorbitol (Kobayashi et al., 2002). Interestingly, this pathway has been observed to occur during both epididymal sperm maturation in humans, cattle, and mice (Frenette et al., 2004, 2006; Jagoe et al., 2013) and conceptus peri-implantation period in pigs (Steinhauser et al., 2016). On the other hand, aldo-keto reductase enzymes have also been found to be implicated in catalyzing the reductive detoxification of carbonyl compounds within the genital tract of rat males (Kobayashi et al., 2002; Iuchi et al., 2004) and in prostaglandin 2 α synthesis (PGF2α) in pigs and humans (Bresson et al., 2011; Seo et al., 2014). As previous studies reported that AKR1B1 is expressed in the endometrium of humans (Bresson et al., 2011) and pigs (Seo et al., 2014) and is involved in PGF2α synthesis, it has been suggested that it could ultimately modulate conceptus implantation through regulation of endometrial gene expression in mammals (Kennedy et al., 2007; Seo et al., 2014). Finally, and reinforcing the belief that this protein plays a major role in reproduction, an in-depth proteomic analysis of pig seminal plasma (SP) revealed that AKR1B1 is positively related to *in vivo* fertility outcomes (Pérez-Patiño et al., 2018).

Seminal plasma, a complex fluid composed of secretions from the testis, epididymis, and male accessory sex glands, mixes with sperm upon ejaculation (Garner and Hafez, 2000). Although the classical roles attributed to SP are acting as a vehicle and serving as a nourishment media for sperm (Garner and Hafez, 2000), mounting evidence indicates that it plays a vital role for sperm function and modulates their fertilizing ability (Rodríguez-Martínez et al., 2011; Locatello et al., 2013). Moreover, SP also interacts with the female reproductive tract modulating the immune environment, a critical point required for successful pregnancy (O’Leary et al., 2004; Robertson, 2007; Schjenken and Robertson, 2014). For this reason, much research has focused on exploring the composition of SP, pointing to some SP-components as potential biomarkers of (in) fertility in several mammalian species (Novak et al., 2010; Milardi et al., 2013; Muhammad Aslam et al., 2014; Cazaux Velho et al., 2018; Pérez-Patiño et al., 2018; Leahy et al., 2019). In this regard, AKR1B1 in SP would be a clear candidate for a fertility biomarker due to its proven relationship with *in vivo* fertility (Pérez-Patiño et al., 2018). However, the mechanism through which SP-AKR1B1 could positively affect fertility in mammals remains unclear.

While the presence of AKR1B1 alongside the female reproductive system of several mammalian species has been extensively reported (Bresson et al., 2011; Seo et al., 2014; Steinhauser et al., 2016), the information about its role in the male genital tract and sperm physiology is scarce. Moreover, although AKR1B1 has been related to epididymal maturation in cattle (Frenette et al., 2004, 2006; Girouard et al., 2009) and mouse (Jagoe et al., 2013), the exact protein synthesis site in the male reproductive system is yet to be reported. In this sense, fractional emission of pig ejaculate offers a valuable opportunity to explore the contributions of specific male sex accessory glands to SP composition (Rodríguez-Martínez et al., 2009). Three fractions/portions can be objectively collected with clear differences in the SP source: (a) the first 10 mL of the so-called sperm rich fraction (SRF-P1), rich in sperm and with SP mainly originating from the epididymis; (b) the rest of SRF (SRF-P2), also rich in sperm and with SP mainly coming from the prostate (Rodríguez-Martínez et al., 2005, 2009), and, (c) the post-SRF (PSRF), poor in sperm and with SP mainly arising from the seminal vesicles (Einarsson, 1971; Rodríguez-Martínez et al., 2005, 2009; Saravia et al., 2009; Rodríguez-Martínez et al., 2011). Interestingly, the SP from these ejaculate portions has also been found to vary in terms of proteomic, metabolomic, and antioxidant capacity (Saravia et al., 2009; Rodríguez-Martínez et al., 2011; Barranco et al., 2015, 2016, 2017; Perez-Patiño et al., 2016; Mateo-Otero et al., 2020).

Considering the well-described relationship of SP-AKR1B1 with *in vivo* fertility outcomes in pigs (Pérez-Patiño et al., 2018) and the multiple key roles of AKR1B1 in reproductive physiology, the overall aim of the present study was to characterize the synthesis of SP-AKR1B1 alongside the male genital tract and to evaluate its involvement in sperm function. To this end, the specific objectives were: (1) to evaluate the concentration of SP-AKR1B1 in different pig ejaculate fractions; (2) to describe the immunohistochemical localization of AKR1B1 alongside the boar reproductive system; and (3) to evaluate the relationship between SP-AKR1B1 and sperm quality/functionality parameters in semen samples (sperm morphology, motility, viability, intracellular H_2_O_2_ production, acrosome integrity, and lipid disorder of plasma membrane). These sperm variables were assessed upon ejaculate collection and after 72 h of storage at 17°C, as liquid storage for that period of time is the most common method for preserving pig semen prior to conducting AI (Kumaresan et al., 2009; Waberski et al., 2019).

## Materials and Methods

### Animals and Samples

All samples were supplied by an Artificial Insemination (AI) Center of AIM Ibérica located in Calasparra (Murcia, Spain), which fulfills the Spanish (ES300130640127; August 2006) and European (ES13RS04P; July 2012) legislation on commercialization of pig semen and animal health and welfare. Samples (ejaculates and reproductive tissues) were obtained from healthy and sexually mature boars (aged 18–36 months) from different breeds and crossbreeds (Pietrain and Duroc). Boars were housed individually in a building with controlled conditions of air temperature (15–25°C) and light (16 h per day), were fed with a commercial diet according to the nutritional requirements of adult boars (Chiba, 2009), and had *ad libitum* access to water.

Sperm quality of each ejaculate used in the experiment was assessed immediately after ejaculation following the standard procedures used in the AI center. All samples fulfilled the standards of sperm number and quality thresholds for the preparation of semen doses used for AI, specifically, (i) more than 200 × 10^6^ spermatozoa/mL, (ii) more than 70% of motile sperm, and (iii) more than 75% of morphologically normal sperm.

Boars were slaughtered in a commercial slaughterhouse (La Mata de los Olmos, Teruel, Spain) for genetic replacement reasons while they were still healthy and suitable as semen donors. Once slaughtered, tissue samples (1 cm × 1 cm and 1 mm thick) from testis, epididymis, and accessory sex glands were collected. Fresh (for immunoblotting analysis) or fixed (4% phosphate-buffered formalin for immunohistochemical analysis) tissue samples were immediately frozen in liquid nitrogen.

### Experimental Design

#### Experiment 1: SP-AKR1B1 Concentration in Ejaculate Portions

For the study of SP-AKR1B1 concentration in ejaculate fractions, the three fractions/portions (SRF-P1, SRF-P2, and PSRF) of seven ejaculates were collected separately, using the gloved hand method. The concentration of AKR1B1 in the SP of each portion was assessed using a porcine-specific enzyme-linked immunosorbent assay (ELISA), as described below.

#### Experiment 2: Expression of AKR1B1 in Male Reproductive Organs

Immunohistochemical and targeted immunoblotting analyses were conducted to find out which organs of the male reproductive system secreted AKR1B1. The samples, from three boars, came from medial testis; caput, corpus, and cauda segments of epididymis; and mid-areas of the prostate, seminal vesicles and bulbourethral glands.

#### Experiment 3: Relationship Between SP-AKR1B1 and Sperm Quality/Functionality Parameters

Entire ejaculates from 14 boars (one ejaculate per boar) were collected using a semi-automatic collection method (Collectis^®^, IMV Technologies, L’Aigle, France) and split into three aliquots. The first aliquot was used for sperm concentration and morphology assessment. The second one was extended alike AI-dose (30 × 10^6^ sperm/mL in Biosem+ extender, Magapor, Zaragoza, Spain) and used to evaluate sperm quality and functionality parameters (sperm motility and viability, intracellular H_2_O_2_ production by viable sperm, and acrosome damage and plasma membrane lipid disorder of viable sperm) after ejaculate collection (0 h) and after 72 h of liquid storage at 17°C. Finally, the third aliquot was centrifuged to obtain SP. Next, SP samples were stored at −80°C until the concentration of AKR1B1 was analyzed with an ELISA assay.

### Seminal Plasma Processing and Storage

Immediately after ejaculate collection, semen samples were centrifuged twice at 1,500 *g* and room temperature for 10 min (Rotofix 32A, Hettich Centrifuges UK, Newport Pagnell, Buckinghamshire, United Kingdom), following a previously described protocol (Perez-Patiño et al., 2016; Li et al., 2018; Barranco et al., 2019; Padilla et al., 2020). After the second centrifugation cycle, the supernatant was examined under a microscope (Eclipse E400, Nikon, Melville, NY, United States) to verify that it was sperm-free. Then, SP samples were split into cryotubes and stored at −80°C (Ultra Low Freezer, Haier Inc., Qingdao, China) until analysis. Samples were thawed on ice prior to evaluation.

### Immunoblotting

In order to lysate tissue samples, 50 mg of each tissue was resuspended in 800 μL of lysis buffer (xTractor^®^ Buffer; Takara Bio, Mountain View, CA, United States) supplemented with 50 U DNase I (Takara Bio), 1% protease inhibitor cocktail, and 700 mM sodium orthovanadate. After incubation at 4°C for 10 min, samples were disrupted mechanically four times using a TissueLyzer II (Qiagen, Hilden, Germany) set at 30 strokes/s for 5 min at 4°C. Subsequently, centrifugation at 12,000 *g* and 4°C for 30 min was carried out in order to obtain the supernatants, which were finally collected and stored at −80°C. Finally, total protein was quantified in triplicate by a detergent compatible (DC) method (BioRad).

For each tissue sample, 10 μg of total protein was diluted in 10 μL of miliQ water. Then, 10 μL of Laemmli reducing buffer 2× supplemented with 5% (v/v) β-mercaptoethanol (BioRad) was added to samples and boiled at 95°C for 5 min. Following this, a total volume of 20 μL per sample was loaded onto a polyacrylamide gradient (8–16%) gel (Mini-PROTEAN^®^ TGX Stain-FreeTM Precast Gels, Bio-Rad); electrophoresis ran at 120–150 V for approximately 1 h. After electrophoresis, total protein content was visualized by UV exposition and acquisition using a G:BOX Chemi XL system (SynGene, Frederick, MD, United States). Afterward, proteins from the resulting gels were transferred onto polyvinylidene difluoride (PVDF) membranes using Trans-Blot^®^ Turbo^TM^ (BioRad). Next, membranes were blocked in blocking buffer (10 mmol/L Tris, 150 mmol/L NaCl, 0.05% Tween-20, and 5% bovine serum albumin; pH = 7.3) (Roche Diagnostics, S.L., Basel, Switzerland) at room temperature for 1 h under agitation. One of the blocked membranes was incubated with the anti-AKR1B1 primary antibody (ref. HPA026425, Prestige Antibodies; Merck, Germany) diluted in blocking solution (1:2,000; v:v), and the other membrane with the same primary antibody (1:2000, v:v) and its blocking peptide (ref. APREST77862, Prestige Antibodies; Merck) 20 times more concentrated than the antibody. Both membranes were incubated at 4°C overnight. Next, membranes were washed three times with TBS-Tween 20 1× (10 mmol/L Tris, 150 mmol/L NaCl, and 0.05% Tween-20; pH = 7.3) for 5 min before incubation with an anti-rabbit, secondary antibody conjugated with HRP (ref. P0448; Sigma Aldrich) diluted in blocking solution (1:3,000; v:v). Membranes were washed 10 times and finally revealed using a chemiluminescent substrate (Immobilion^TM^ Western Detection Reagents, Millipore); images were scanned with G:BOX Chemi XL 1.4.

### Immunohistochemistry

The sections of paraffin-embedded tissue samples (male reproductive organs and liver as a positive control) were immunohistochemically stained using an avidin-biotin complex protocol (Vector Laboratories, Burlingame, CA, United States). Briefly, sections were first deparaffinized two times using Histo-Clear II (Electron Microscopy Sciences, Hatfield, England) and progressively rehydrated through a decreasing ethanol series from 100 to 70% and distilled water. Next, and in order to allow antigen retrieval, sections were microwaved four times in 10 mM Tris-1 mM EDTA buffer (pH = 9.0) for 5 min, with intermediate refiling with Tris-EDTA. Then, sections were washed with tap water and placed in racks. Samples were incubated with a blocking and permeabilizing solution composed of 3% bovine serum albumin (BSA) in 0.1% PBS-Tween at room temperature for 30 min. Afterward, sections were incubated overnight at 4°C with the rabbit anti-AKR1B1 primary antibody diluted 1:100 (v:v) in PBS with 1% Triton X-100 containing 3% BSA. As a negative control, the primary antibody was omitted. On the other hand, in order to prove antibody specificity, samples were incubated with the AKR1B1 primary antibody and its blocking peptide, which was 50 times in excess. The next day, sections were washed and subsequently incubated with a polyclonal goat anti-rabbit secondary antibody conjugated with biotin (EDM Millipore Corporation, Temecula, CA, United States) diluted 1:200 (v:v) in PBS containing 1% Triton X-100 and 3% BSA at room temperature for 30 min. Sections were then washed and incubated with 3% H_2_O_2_ in BSA-PBS for 20 min to block endogenous peroxidase activity. Next, all sections were incubated with the VECTASTAIN ABC reagent (Vector Laboratories, Burlingame, CA, United States) for 1 h, and with DAB peroxidase substrate working solution (Vector Laboratories, Burlingame, CA, United States) for 10 min. Slides were counterstained with Harris hematoxylin (Thermo Fisher Scientific, Waltham, MA, United States), dehydrated with an increasing ethanol series, and mounted with Eukitt^®^ mounting medium (PanReac, Barcelona, Spain). Finally, slides were microscopically evaluated and photographed using Nikon Eclipse EP2000-S (Nikon).

### Enzyme-Linked Immunosorbent Assay

Concentration of AKR1B1 in SP was quantified using a porcine-specific quantitative sandwich ELISA kit (MBS9316209; MyBioSource, San Diego, CA, United States) following the manufacturer’s manual. Briefly, to obtain the standard curve, 50 μL of AKR1B1 standards (0.625, 1.25, 2.5, 5, 10, and 20 ng/mL) was added to the plate in duplicate. On the other hand, SP samples were thawed, diluted in PBS 1× (1:75; v:v) and added to the plate in duplicate. The content of all wells, except the blank ones, was mixed with 100 μL of HRP-Conjugate Reagent, and the plate was subsequently incubated at 37°C for 60 min. After washing all wells four times, 50 μL of Chromogen A and 50 μL of Chromogen B were added. After mixing gently, the plate, protected from light, was incubated at 37°C for 15 min. Next, 50 μL of the Stop solution was added to all wells and, after 5 min, the plate was read at 450 nm using a microplate spectrophotometer (BioTek Epoch; BioTek, Winooski, Vermont, United States). The average of the duplicate reading for each standard was calculated and the average optical density from the blank was subtracted. Based on AKR1B1 standards, a linear regression curve interpolating AKR1B1 concentration from absorbance reading was calculated; the equation resulted to be: [AKR1B1] = Abs+0.038/0.0522, *R*^2^ = 0.9772.

This ELISA kit was highly specific for porcine AKR1B1, showing a sensitivity of 0.1 ng/mL and a detection range of 0.625–20 ng/mL.

### Sperm Quality and Functionality Parameters’ Assessments

For evaluation of sperm quantity and functionality, seven parameters were assessed: (i) concentration, (ii) morphology, (iii) motility, (iv) viability, (v) intracellular H_2_O_2_ production by viable sperm, (vi) acrosome damage in viable sperm, and (vii) plasma membrane lipid disorder in viable sperm. Except for sperm concentration, which was only evaluated immediately after ejaculate collection (0 h), the other quality/functionality variables were determined at two time-points: after ejaculate collection (0 h) and after 72 h of liquid storage at 17°C (72 h).

For sperm concentration assessment, a high-precision automated cell counter (NucleoCounter^®^NC-100^TM^, ChemoMetec, Allerod, Denmark) was used following manufacturer’s recommendations. Sperm morphology was examined under a phase-contrast microscope at 1,000× magnification (Nikon Labophot, Nikon, Tokio, Japan) in semen samples diluted (1:1; v:v) with 0.12% formaldehyde saline solution (Panreac). A total of 200 sperm cells were counted and classified as morphologically normal if they did not exhibit abnormal head, acrosome defects, proximal cytoplasmic droplets, distal cytoplasmic droplets, folded tails, or coiled tails. Sperm motility was assessed using a computer-assisted sperm analyzer (CASA, ISASV1^®^, Proiser R+D S.L., Paterna, Spain). For this analysis, 5 μL of each semen sample (30 × 10^6^ sperm/mL in Biosem+) was pipetted onto a pre-warmed (38°C) Makler chamber (Sefi Medical Instruments, Haifa, Israel). A total of 10 different fields per sample accumulating ≥600 sperm were acquired and examined. For further analysis, percentages of total motile sperm (sperm showing an average path velocity ≥20 μm/s) and progressively motile sperm (exhibiting rapid and progressive movement with a straight-line velocity ≥40 μm/s) were recorded.

Sperm viability, acrosome damage, intracellular H_2_O_2_ production, and membrane lipid disorder were assessed by flow cytometry (BD FACS Canto II; Becton Dickinson & Company, Franklin Lakes, NJ, United States). For each semen sample and sperm parameter, three technical replicates with 10,000 Hoechst 33342 (H-42; Merck)-positive events were evaluated.

Sperm viability and acrosome damage were assessed using a triple-staining with Hoechst 33342 (H-42), propidium iodide (PI; Merck), and fluorescein-conjugated peanut agglutinin (PNA-FITC; Merck). Briefly, 100 μL of each semen sample (30 × 10^6^ sperm/mL in Biosem+) was incubated with 3 μL H-42 (0.05 mg/mL in PBS), 2 μL PI (0.5 mg/mL in PBS), and 2 μL PNA-FITC (100 μg/mL in PBS) at 37°C (Sanyo MIR-153 incubator, Gemini BV, Apeldoorn, Netherlands) for 10 min. Next, 400 μL PBS was added to each sample. Percentages of viable spermatozoa (H-42+/PI-) with an intact (PNA-FITC-) and non-intact (PNA-FITC+) acrosome membrane were recorded.

To assess intracellular H_2_O_2_ production by viable sperm, a triple-staining with H-42, PI, and 5- and 6-chloromethyl-2′,7′-dichlorodihydrofluorescein diacetate acetyl ester (CM-H_2_DCFDA; Merck) was prepared. Briefly, 50 μL of each semen sample (30 × 10^6^ sperm/mL in Biosem+) was incubated with 1.5 μL H-42 (0.05 mg/mL in PBS 1×), 1 μL PI (0.5 mg/mL in PBS), and 1 μL CM-H_2_DCFDA (1 mM in dymetilsulfoxide [DMSO]) in 950 μL PBS at 37°C for 30 min. An aliquot of each semen sample was incubated with all fluorochromes plus 1 μL of tert-butyl hydroperoxide solution (70% in distilled water) and was used as a positive control. The percentage of viable sperm (H-42+/PI−) that exhibited high intracellular H_2_O_2_ generation (2′,7′-di-chlorofluorescein [DCF]+) was recorded.

Finally, to evaluate the lipid disorder of plasma membrane in viable sperm, a triple-staining with H-42, Yo-Pro-1 (Merck), and Merocyanine 540 (M-540; Merck) was carried out. Briefly, 50 μL of each semen sample (30 × 10^6^ sperm/mL in Biosem+) was incubated with 2.5 μL H-42 and 10 μL Yo-Pro-1 (2.5 μM in DMSO) in 950 μL PBS at 37°C for 8 min. After this period, 26 μL of M540 (0.1 mM in DMSO) was added to each sample and incubated at 37°C for 2 min. The percentage of viable spermatozoa (H-42+/Yo-Pro-1-) exhibiting lipid membrane disorder (M-540+) was recorded.

### Statistical Analysis

Data were analyzed using the statistical package IBM SPSS 25.0 for Windows (IBM corp., Armonk, NY, United States). First, normal distribution was tested with Shapiro-Wilk test and homogeneity of variances was checked with Levene test. Concentrations of AKR1B1 between the three SP portions (i.e., SRF-P1, SRF-P2, and PSRF) were compared through one-way analysis of variance (ANOVA) followed by *post hoc* Sidak test. Ejaculates were classified based on their AKR1B1 concentration into groups through a two-step cluster analysis using the log-likelihood distance and the Schwarz’s Bayesian criterion. Following this, sperm quality and functionality variables (sperm motility and viability, intracellular H_2_O_2_ production by viable sperm, acrosome damage in viable sperm, and plasma membrane lipid disorder in viable sperm) were compared between the two groups of ejaculates (high SP-AKR1B1 and low AKR1B1) with a linear mixed model followed by *post hoc* Sidak test. In this model, between-subjects factor was the ejaculate group and within-subjects factor was the time of semen storage at 17°C. When needed, data were linearly transformed with arcsine √x. The level of statistical significance was set at *P* ≤ 0.05.

## Results

### Experiment 1: SP-AKR1B1 Concentration in Ejaculate Portions

**FIGURE 1:**
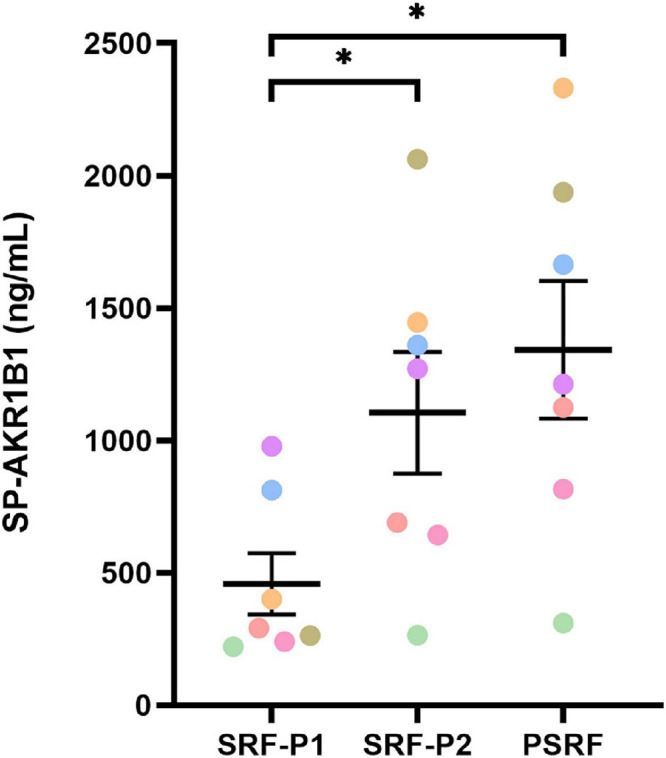
Seminal plasma-AKR1B1 concentration (ng/mL) in the different pig ejaculate fractions: the first 10 mL of the sperm rich fraction (SRF-P1), the rest of the sperm rich fraction (SRF-P2), and post sperm rich fraction (PSRF). Data are represented as mean ± standard error of the mean. Different dot colors represent each individual (*n* = 7). Differences between fractions are indicated as * (*P* ≤ 0.05).

Concentrations of AKR1B1 in SP fractions/portions are shown in Figure 1. The SP from SRF-P1 from SRF-P1 exhibited the lowest (*P* < 0.05) AKR1B1 concentration (458.2 ± 116.33 ng/mL) compared to SRF-P2 (1105.0 ± 229.80 ng/mL) or PSRF (1342.4 ± 260.18 ng/mL). Moreover, no differences (*P* > 0.05) in AKR1B1 concentrations were found between SRF-P2 and PSRF. Finally, no breed effect was observed in SP-AKR1B1 concentration, as Pietrain and Duroc boars showed similar concentrations (522.2 ± 66.85 ng/mL vs. 691.7 ± 81.00 ng/mL; *P* > 0.05).

### Experiment 2: Expression of AKR1B1 in Male Reproductive Organs

**FIGURE 2:**
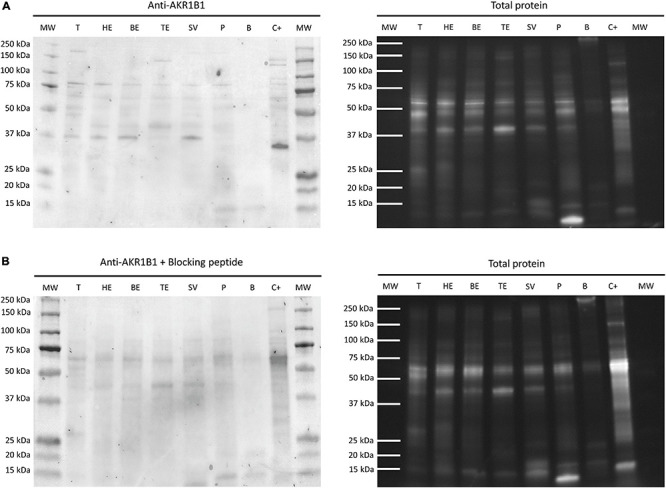
Representative Western blot of **(A)** anti-AKR1B1 and **(B)** its corresponding blocking peptide, and their total protein controls in boar reproductive tissues. MW, molecular weight; T, testis; HE, caput epididymis; BE, corpus epididymis; TE, cauda epididymis; SV, seminal vesicles; P, prostate; B, bulbourethral glands; C+, liver.

The immunoblotting assay revealed the presence of AKR1B1 along the entire male reproductive tissues except for bulbourethral glands (Figure 2). Specifically, two specific bands were detected: (i) a 36 kDa band was found in testis, epididymal caput and corpus, and seminal vesicles; and (ii) a ∼80 kDa band was also identified in testis, cauda, caput and corpus of epididymis, seminal vesicles, and prostate. Both bands also appeared in the positive control (liver). The two bands were not seen when membranes were incubated with AKR1B1 blocking peptide, revealing that they were specific for AKR1B1. Therefore, bands of ∼40 and ∼70 kDa should be considered as unspecific for this antibody.

**FIGURE 3:**
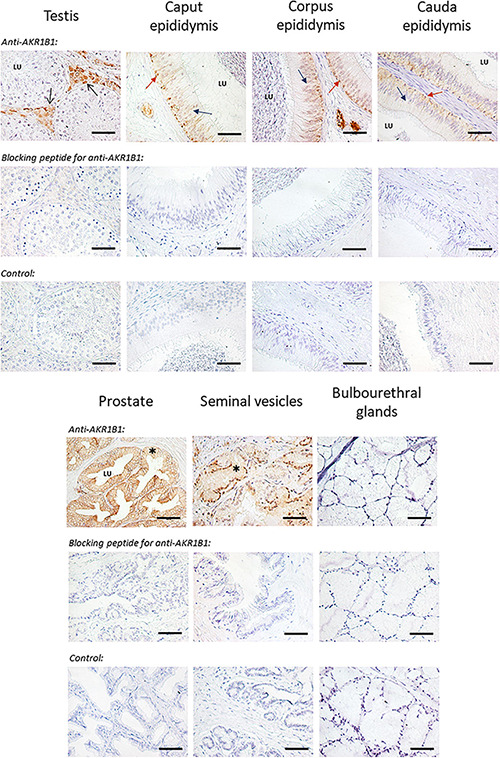
Immunohistochemistry of AKR1B1 in boar genital organs (testis, caput, corpus, and cauda from the epididymis, prostate, seminal vesicles, and bulbourethral glands). The first row shows anti-AKR1B1 staining in each tissue. Specifically, Sertoli cells from testis (black arrow), principal (blue arrow), and basal cells (orange arrow) in the epididymis and glandular cells from prostate and seminal vesicles (indicated as *) appeared stained. Specificity of the primary antibody in all tissues/organs was confirmed by the absence of signal in blocking peptide assays (second row). Finally, negative control (third row) proved the specificity of the secondary antibody. LU, lumen. Scale bar = 50 μm.

The AKR1B1 protein was immunohistochemically detected in the reproductive tissues analyzed (Figure 3). Two controls were used: (i) the specificity of the primary antibody was confirmed in all tissues through incubation with the AKR1B1 primary antibody with blocking peptide and (ii) the specificity of the secondary antibody was proven with the omission of the primary antibody in all tissue samples. The presence of AKR1B1 was confirmed in all reproductive tissues except for bulbourethral glands, in which no staining was observed. In testis, AKR1B1 was observed in the interstitial tissue, specifically in the cytosol of Leydig cells, whereas no presence of the protein was detected in seminiferous tubules. Regarding epididymis, AKR1B1 was localized in basal and principal cells of the epithelia of all regions (caput, corpus, and cauda). In addition, both prostate and seminal vesicles showed cytoplasm immunostaining in glandular epithelial cells. Finally, AKR1B1 was undetectable in bulbourethral glands.

### Experiment 3: Relationship Between SP-AKR1B1 and Sperm Quality and Functionality Parameters

**FIGURE 4:**
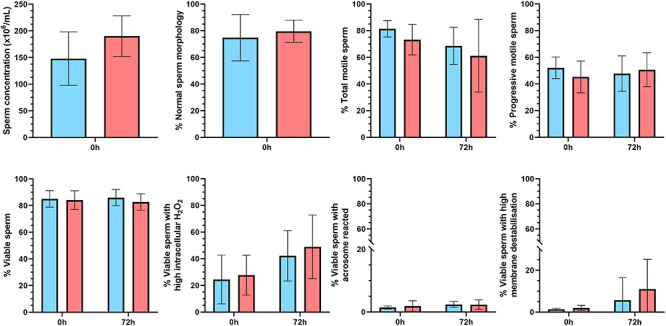
Sperm quality and functionality parameters upon semen collection (0 h) and after 72 h of liquid storage at 17°C in two groups ejaculates clustered on the basis of AKR1B1 concentration in seminal plasma (SP) samples. Fourteen ejaculates were classified into two groups: with low SP-AKR1B1 levels (ranging from 376.4 to 756.3 ng/mL, *n* = 7; blue bars) and with high SP-AKR1B1 levels (ranging from 842.2 to 1211.25 ng/mL, *n* = 7; red bars). Data are represented as mean ± standard error of the mean.

In order to evaluate the relationship between SP-AKR1B1 and sperm quality and functionality parameters, 14 ejaculates were classified (hierarchical clustering; *P* < 0.001) into two groups: with low SP-AKR1B1 (ranging from 376.4 to 756.3 ng/mL, *n* = 7) and high SP-AKR1B1 levels (ranging from 842.2 to 1211.25 ng/mL, *n* = 7; Figure 4). No differences (*P* > 0.05) in any of the different sperm quality and functionality parameters assessed (sperm concentration, normal sperm morphology, total and progressive motility, viable sperm, viable sperm with high intracellular H_2_O_2_, viable sperm with a damaged acrosome, and viable sperm with high membrane destabilization) were observed between high and low SP-AKR1B1 groups at any evaluation time-point (0 and 72 h of storage at 17°C).

## Discussion

To the best of our knowledge, this is the first study characterizing the expression of AKR1B1 along the male reproductive system in livestock. Likewise, this report is also the first relating the concentration of AKR1B1 in SP with sperm quality and functionality parameters of liquid-stored semen samples. Specifically, the results showed that: (i) monomeric and dimeric AKR1B1 forms were expressed in all male reproductive tissues, except bulbourethral glands; (ii) AKR1B1 was expressed in Sertoli cells, basal and principal epididymal cells, and glandular cells from the prostate and seminal vesicles; (iii) seminal vesicles were likely to contribute the most to the final SP-AKR1B1 concentration of the pig ejaculate; and (iv) SP-AKR1B1 levels were not related to sperm quality and functionality parameters, nor was this enzyme involved in the sperm resilience to preservation at 17°C for 72 h.

Characterization of SP components, including proteins or even metabolites, has been a source of sperm quality, functionality, and fertility biomarkers in several mammalian species (Milardi et al., 2013; Cazaux Velho et al., 2018; Moura et al., 2018; Pérez-Patiño et al., 2018). In this sense, as mentioned before, AKR1B1 has been reported to have multiple roles in the reproductive physiology of humans (Bresson et al., 2011), cattle (Frenette et al., 2004; Girouard et al., 2009), rodents (Kobayashi et al., 2002), and pigs (Steinhauser et al., 2016; Pérez-Patiño et al., 2018). However, no information regarding its synthesis along the male reproductive tract exists in any mammalian species.

The immunoblotting and immunohistochemistry results showed that AKR1B1 was expressed in testis, epididymis, and all accessory sex glands, except the bulbourethral ones. These results are in agreement with observations in male rats, in which the activity of aldose reductase was observed along all the male reproductive tract, except in bulbourethral glands that were not analyzed (Kobayashi et al., 2002; Iuchi et al., 2004). Moreover, other studies conducted in cattle and pigs demonstrated the presence of AKR1B1 in seminal vesicles (Samuels et al., 1962; Westfalewicz et al., 2017). This protein has been reported to interact with epididymal sperm during maturation (Frenette et al., 2003, 2006; Katoh et al., 2014), which would advise a role of AKR1B1 in sperm physiology. In this context, the presence of AKR1B1 has been proposed to contribute to the acquisition of sperm motility and fertilizing ability in pigs (Katoh et al., 2014), as well as to support bovine sperm survival during epididymal transit and storage (Frenette et al., 2003). In addition, our immunoblotting assay showed the presence of one or two specific bands (36 and ∼80 kDa) in most male reproductive tissues. While dimerization of AKR1B1 has been previously reported in ovine thymus (Yang et al., 2019) and bovine peripheral blood mononuclear cells (Yang et al., 2016), there are no previous studies of such a dimerization in SP samples from any mammalian species. Indeed, as monomeric and dimeric protein forms are known to have different cellular functions, further studies should address whether these forms could explain the different roles of SP-AKR1B1 in reproductive physiology.

Besides the presence of AKR1B1 along the male reproductive tract, our study found that this protein was only present in specific cell types, rather than in the lumen of the different organs. Specifically, in testis, only Leydig cells were stained with the anti-AKR1B1 antibody. Since these cells are implicated in hormonal secretion and spermatogenesis regulation (Zhou et al., 2019), one could hypothesize that AKR1B1 is involved in these processes. On the other hand, AKR1B1 was also found in both basal and principal cells along the entire epididymis and glandular cells from the prostate and seminal vesicles. Considering that all of these cell types are involved in protein secretion (Leung et al., 2004; Breton et al., 2019), the presence of AKR1B1 in SP can be assumed to be originated from the collective synthesis and secretion of the aforementioned organs. These findings are in agreement with a previous study conducted in bovine, in which aldose reductase was also reported to be present in the same epididymal and testicular cells lines (Frenette et al., 2003). However, this is the first report in a mammalian species describing that vesicle glands are the main synthesis site. Nevertheless, to the best of our knowledge, neither the exact contribution of the different male accessory glands to AKR1B1 levels in SP, nor its potential role on ejaculated sperm has been uncovered.

ELISA assay of the different ejaculate portions confirmed immunohistochemical and immunoblotting results, indicating that whereas AKR1B1 was present in the SP from all ejaculate portions, a higher concentration of this protein was found in the SP from PSRF. As aforementioned, SP from each ejaculate portion has a different origin: SP from SRF-P1 is mainly secreted by the epididymis, SP from SRF-P2 originates from the epididymis and prostate, and SP from PSRF is mainly produced by seminal vesicles (Einarsson, 1971; Rodríguez-Martínez et al., 2009; Saravia et al., 2009; Rodríguez-Martínez et al., 2011). Considering these results, one could hypothesize that seminal vesicles are the principal contributor for SP-AKR1B1 secretion to the entire ejaculate. Similar results have been reported in bovine, where this enzyme is one of the most abundant proteins of the seminal vesicle fluid (Westfalewicz et al., 2017).

The present study also evaluated the relationship between SP-AKR1B1 concentration and sperm quality and functionality after both 0 and 72 h of liquid storage at 17°C. No relationship between SP-AKR1B1 concentration and any of the evaluated parameters, which included sperm concentration, normal sperm morphology, total and progressive motility, sperm viability, and percentages of viable sperm with high intracellular ROS, viable sperm with a damaged acrosome, and viable sperm with high membrane destabilization, was observed. To the best of our knowledge, there is scarce information regarding the role of AKR1B1 in sperm physiology. While AKR1B1 has been reported to be overexpressed in men with high seminal lipid peroxidation levels (Intasqui et al., 2015) and to be involved in porcine sperm capacitation (Katoh et al., 2014), none of these parameters were evaluated in the present study. Thus, taking into account the lack of influence of SP-AKR1B1 on sperm quality and functionality found in our study, together with the fact that the highest SP-AKR1B1 concentration was not found in the SRF (which contains most of the ejaculated sperm; Einarsson, 1971; Rodríguez-Martínez et al., 2009; Saravia et al., 2009; Rodríguez-Martínez et al., 2011), it is reasonable to surmise that SP-AKR1B1 does not play a major role on the sperm quality and functionality parameters assessed. However, this protein could play a crucial role on sperm lipid peroxidation or during capacitation. Further studies are required to evaluate this potential relationship.

Besides its relevance during epididymal maturation (Frenette et al., 2003; Katoh et al., 2014), SP-AKR1B1 could have a potential influence on the female reproductive tract, as it is expressed in the endometrium in humans (Chapdelaine et al., 2006; Bresson et al., 2011) and pigs (Seo et al., 2014; Steinhauser et al., 2016). In the uterus, the luminal AKR1B1 has been found to be involved in the polyol pathway during the conceptus peri-implantation period and, in porcine, its expression is downregulated as conceptus attaches to the endometrium (Steinhauser et al., 2016). On the other hand, uterine AKR1B1 is involved in progesterone synthesis in humans (Bresson et al., 2011) and pigs (Seo et al., 2014), thus contributing to prepare the endometrium for conceptus implantation as well as modulating the maternal immune system (Czyzyk et al., 2017). Our results, together with the fact that SP-AKR1B1 is linked with high *in vivo* pregnancy outcomes (Pérez-Patiño et al., 2018), suggest that SP-AKR1B1 could act jointly with the endometrial AKR1B1 to prepare the uterine environment for conceptus implantation. Another possibility could be that this protein has a direct impact on conceptus. In this sense, SP has been reported to exert a positive effect on pregnancy outcomes, specifically improving implantation and pregnancy rates in humans (Crawford et al., 2015; Saccone et al., 2019) and even modifying embryo gene expression in pigs (Martinez et al., 2020). Thus, it cannot be discarded the influence of SP-AKR1B1 on the improvement of embryo survival and even implantation, through the modulation of the uterine environment. In this regard, all these functions could be driven by both the soluble form of the protein in SP or by that contained in extracellular vesicles (EVs). SP EVs regulate sperm function through its integration to sperm membrane (Leahy et al., 2020) and their action on the female immune system (Zhang et al., 2020). Interestingly, bovine AKR1B1 has been reported to be associated to epididymal EVs (Frenette et al., 2006) and, in humans, seminal EVs have been found to contain aldose reductase (Zhang et al., 2020). In pigs, however, whether this protein is also in the cargo of SP EVs and participates in fertilization or embryo development remains to be explored.

AKR1B1 has been extensively demonstrated to play a role in female and male reproductive physiology. In pigs, SP-AKR1B1 has been reported to exert a positive impact on *in vivo* fertility outcomes. This study demonstrated that all male genital organs (except bulbourethral glands) are able to express AKR1B1. We also found that the concentration of AKR1B1 was higher in the post-SRF, suggesting that seminal vesicles could be the main contributor of this SP protein to the final ejaculate. Our results also indicated that SP-AKR1B1 is not associated to the quality and functionality parameters of sperm. These findings, together with the fact that this protein has been shown to be positively related to *in vivo* fertility (Pérez-Patiño et al., 2018), suggest that it could play an active role in the female reproductive tract, promoting sperm fecundity or even embryo development. Thus, further studies to determine the exact mechanism through which SP-AKR1B1 has a positive influence on *in vivo* fertility outcomes should be conducted.

## Data Availability Statement

The raw data supporting the conclusions of this article will be made available by the authors, without undue reservation.

## Author Contributions

YM-O, MY, and IB: conceptualization. YM-O, EV-V, and ML: methodology. YM-O, JR-M, MY, and IB: formal analysis and investigation. YM-O and EV-V: writing—original draft preparation. JR-M, JR, MY, and IB: writing—review and editing. JR and MY: funding acquisition. IB and MY: supervision. All authors read and agreed to the published version of the manuscript.

## Conflict of Interest

The authors declare that the research was conducted in the absence of any commercial or financial relationships that could be construed as a potential conflict of interest.
